# A Study of Hypomagnesemia in Patients Admitted to the ICU

**DOI:** 10.7759/cureus.41949

**Published:** 2023-07-16

**Authors:** Kakarlapudi Santosh Raju, Janapareddi V BhaskaraRao, Bobbili Tarun Kesava Naidu, Nallapati Sunil Kumar

**Affiliations:** 1 General Medicine, Maharajah's Institute of Medical Sciences (MIMS), Vizianagaram, IND; 2 Neurology, Gitam Institute of Medical Sciences and Research, Visakhapatnam, IND; 3 General Medicine, Sri Lalithambigai Medical College and Hospital, Chennai, IND

**Keywords:** magnesium levels, morbidity and mortality, critically ill patients, medical icu, hypomagnesimia

## Abstract

Introduction

After potassium, magnesium (Mg2+) is the most prevalent cation found intracellularly in the human body. The maintenance of excitability by Mg2+ and other cations is crucial for the neuromuscular junction to operate normally. Magnesium shortages are frequently overlooked compared to other electrolyte disorders such as hyponatremia, hypokalemia, and hypocalcemia. The present study aimed to study the factors and effects of hypomagnesemia among intensive care unit (ICU) patients who are critically ill at a tertiary care hospital.

Methods

This is a prospective observational study done among the patients who got admitted to the Medical ICU at Maharajahs Institute of Medical Sciences (MIMS), Nellimarla, Vizianagaram, from January 2020 to June 2021. A total of 100 patients were included in the study, of which 50 were cases and 50 were controls. The cases are those patients admitted to the medical ICU with critical illness and hypomagnesemia, and the controls are selected from those patients admitted with critical illness to the medical ICU with normal magnesium levels. Patients with severe infections, including sepsis, respiratory failure, cardiac failure, renal failure, cerebrovascular accidents (CVA), poisonings, and diabetic ketoacidosis (DKA), were included in the study. Patients who were treated with magnesium before admission to our ICU were excluded from this study. Serum magnesium was tested within 24 hours of admission and is correlated with the outcomes of the patients in terms of APACHE II score, length of ICU stay, and requirement and duration of ventilatory support.

Results

Out of a total of 50 cases, 29 (58%) are of males. The mean age of cases was 57.6 ∓ 8.2 years. Most cases were admitted due to organ failure (30%), followed by sepsis (26%) and poisoning (22%). The mean magnesium levels were 1.19 mg/dL among the cases, which was significantly lower when compared to the control group (2.16 mg/dL) (*p*-value= 0.01). The mean length of stay in the ICU was 6.12∓5.16 days in cases, whereas it was 5.28∓3.37 days in the control group (*p-*value = 0.33). 12% of cases needed non-invasive ventilation (NIV) when compared to 8% of controls (*p*-value= 0.50). 48% of the cases needed invasive ventilator support when compared to 28% in the control group (*p-*value= 0.03). The duration of invasive ventilation was higher among the cases (mean = 10 ∓3-17 days) compared to the controls (mean = 3 ∓2-4 days); *p-*value = 0.001. Mortality was higher in the case group at 28% (14) and was 10% (5) in the control group (*p*-value = 0.02).

Conclusion

The need for invasive ventilation and duration of invasive ventilation were significantly higher among the patients with hypomagnesemia compared to the patients with normal magnesium levels (*p-*value <0.05). Mortality was higher in the cases than in the controls (*p-*value <0.05).

## Introduction

In the human body, after potassium, magnesium (Mg2+) is the most abundant cation present intracellularly. It participates as a co-factor mainly in catalytic reactions that involve a shift of the phosphate group. Mg2+, along with other cations, is essential for the normal functioning of the neuromuscular junction as it maintains excitability. Mg2+ is an essential cation that helps maintain cardiac excitation [1]. Hypomagnesemia is seen in 20% to 65% of ICU cases who are critically ill [2]. Magnesium deficiency is commonly overlooked when compared to electrolyte disturbances like hyponatremia, hypokalemia, and hypocalcemia. Hypomagnesemia is frequently seen in association with hypokalemia, and it acts as a masquerader for hypokalemia. The most common causes are decreased absorption from the gastrointestinal tract (GIT), suction from the nasogastric tube, feeding formulas poor in magnesium, and pharmaceutical agents like diuretics and aminoglycosides [3].

The prognosis of ICU patients is affected by hypomagnesemia. Magnesium depletion is associated with increased mortality, morbidity, and ICU and hospital stays. It also increases healthcare costs and burdens our healthcare system [4]. The present study aims to study the effects of hypomagnesemia among ICU patients who are admitted to the ICU in a tertiary care hospital in terms of mortality, duration of hospital stay, and duration and requirement of ventilatory support.

## Materials and methods

This prospective observational study was conducted among critically ill medical patients who were admitted to the Medical Intensive Care Unit (ICU) at Maharajahs Institute of Medical Sciences (MIMS), Nellimarla, Vizianagaram, from January 2020 to June 2021. A total of 100 patients were included in the study, of which 50 were cases and 50 were controls. The cases are those patients admitted to the medical ICU with critical illness and hypomagnesemia, and the age- and sex-matched controls were selected from those patients admitted to the medical ICU with normal magnesium levels. Patients with sepsis, respiratory failure, cardiac failure, renal failure, cerebrovascular accidents (CVA), poisonings, and diabetic ketoacidosis (DKA) were included in the study. Patients treated with magnesium before admission to the ICU were excluded from this study. Other cation deficiencies were treated appropriately in both groups. After admission to the ICU, serum magnesium was tested within 24 hours, repeated every 24 hours, and noted. A serum magnesium value of 1.8-2.2 mg/dL was considered normal. Age, gender, APACHE II score, length of ICU stay, and ventilatory support requirement and duration were analyzed among cases and controls. The initial serum magnesium levels were compared with the final outcomes of the patients in terms of APACHE II score, length of ICU stay, requirement and duration of ventilatory support, and mortality. Institutional ethical committee clearance was obtained from IEC, Maharajah's Institute of Medical Sciences, Vizianagaram (IEC/42/20). After taking informed and written consent from the patients or their attendants, the collected data were entered into MS Excel (Redmond, USA) sheets and analyzed using Microsoft Excel 2019 and IBM Corp. Released 2015. IBM SPSS Statistics for Windows, Version 23.0. Armonk, NY: IBM Corp. Categorical data was represented in the form of frequencies. Continuous data were represented as the mean and standard deviation. A p-value of <0.05 was considered statistically significant. The chi-square test and unpaired t-test were used in drawing the correlations. 

## Results

Out of a total of 50 cases, 29 (58%) were of males, and the rest 21 (42%) were of females. Out of a total of 50 controls, 31 (62%) were of males, and the rest 19 (38%) were of females. The difference in gender distribution is not statistically significant (p = 0.68). In this study, most cases fell under the age group of 61-70 (30%), and the mean age of cases was 57.6 ∓ 8.2 years. The minimum age in the case group was 20 years, and the maximum age in the case group was 84. In the control group, the mean age was 52.52 ∓ 7.5 years, and the maximum and minimum ages were 80 and 18, respectively. The difference between age among cases and controls was not statistically significant (p=0.08). Among the cases, 14 patients out of 50 had diabetes, whereas nine patients out of 50 in controls had diabetes, and the difference is not statistically significant at a p-value of 0.23. Patients in the control group had lower APACHE scores when compared with the case group, i.e., 17 out of 50 (34%) controls had a score of less than four, whereas 15 out of 50 (30%) cases had scores in the range of 10-15. The mean APACHE score in the case group was 11.64, while in the control group, it was 9.5. The difference in APACHE scores between both groups was not significant at a p-value of 0.7. 

The mean magnesium levels were 1.19 mg/dL among the cases, which was significantly lower when compared to the control group (2.16 mg/dL) (p-value= 0.01). The mean length of stay in ICU was 6.12 ∓ 5.16 days in cases, whereas it was 5.28 ∓ 3.37 days in the control group, and the association was statistically not significant with a p-value of 0.33, as shown in Table 1.

**Table 1:** Comparison of length of stay in the ICU between cases and controls

Group	Mean (days)	Standard deviation
Cases	6.12	5.16
Controls	5.28	3.37
P-value	0.33	

12% of cases needed non-invasive ventilation (NIV) when compared to 8% of controls (p-value= 0.50). 48% of the cases needed invasive ventilator support when compared to 28% among the control group (p-value= 0.03), as shown in Table 2.

**Table 2:** Comparison of the need for ventilator support between cases and controls

Ventilator support	Subcategory	Cases	Controls	Total	P-value
Non-invasive	Yes	6 (12%)	4 (8%)	10 (10%)	0.50
	No	44 (88%)	46 (92%)	90 (90%)	
Invasive	Yes	24 (48%)	14 (28%)	38 (38%)	0.03
	No	26 (52%)	36 (72%)	62 (62%)	

The duration of invasive ventilation was higher among the cases (mean = 10 ∓3-17 days) compared to the controls (mean = 3 ∓2-4 days); p < 0.001. In our study, we observed that the incidence of hypokalemia was higher in the case group, 24% (12), while it was 12% (6) in the control group, and the association was statistically not significant with a p-value of 0.11. 12% (6) of cases were associated with hypocalcemia, whereas it was 8% (4) in the control group, and the association was statistically not significant with a p-value of 0.5. Mortality was higher in the case group, 28% (14), and was 10% (5) in the control group. The association was statistically significant, with a p-value of 0.02, as shown in Table 3.

**Table 3:** Comparison of mortality between cases and controls

Mortality	Cases	Controls	Total	P-value
Death	14 (28%)	5 (10%)	19 (19%)	0.02
Survived	36 (72%)	45 (90%)	81 (81%)	
Total	50 (100%)	50 (100%)	100 (100%)	

## Discussion

In humans, magnesium is the second most common intracellular cation. For maintaining the normalcy of homeostasis, Mg2+ plays a vital role. Magnesium serves as a co-factor for most of the adenosine triphosphate (ATP) reactions. Almost 65% of patients admitted to the ICU with critical illnesses develop magnesium deficiency during their stay. Chernow and colleagues reported magnesium deficiency, which is positively correlated with increased mortality in critically ill cases [5]. In healthy people, the serum concentration of magnesium is 0.65 to 1.05 mg/dL. If magnesium excretion rises, its absorption increases by 80% if its intake is low. Important sites for magnesium absorption are the distal small bowel and colon. Magnesium deficiency in critically ill patients is associated with reduced intake, use of TPN solutions (total parenteral nutrition), intracellular magnesium shift in metabolic acidosis, following cardiac bypass, increased losses due to diarrhea, nasogastric route suction, pancreatitis, use of loop diuretics, Amphotericin B, alcoholism, diabetes, hyperthyroidism, and hypercalcemia [6-8]. Clinical effects of hypomagnesemia include cardiac dysrhythmias, prolonged PR and QT intervals, seizures, endocrine disturbances, muscle weakness, and respiratory failure [9]. Mg2+ plays a vital role in the pathophysiology of septicemia. Endothelin and pro-inflammatory cytokine levels are elevated in hypomagnesemia patients. Whang et al. reported the association of hypomagnesemia in 42% of patients with hypokalemia, 29% of patients with hypophosphatemia, 27% of patients with hyponatremia, and 22% of patients with decreased calcium levels [10].

In the present study, we have compared hypomagnesemia patients with normal magnesium concerning morbidity and mortality among ICU patients. Here we have taken a total of 100 patients, which includes 50 cases and 50 control groups. In the present study, the mean magnesium levels were 1.19 mg/dL among the cases, which was significantly lower when compared to the control group, which was 2.16 mg/dL, with a p-value of 0.01, which is statistically significant. Among the ICU scoring systems, the APACHE score is commonly used to prognosticate the patient's condition. The mean APACHE II score in the case group was 12.58, and it was 9.50 in the control group, with a p-value of 0.04. In the Soliman et al. study, they observed that those patients who had ionized hypomagnesemia throughout their ICU period also had higher APACHE scores on admission [11]. Rahul Chowdary Kongara et al. reported that hypomagnesemia is related to an increased mortality rate and a higher APACHE II score, and the relation was statistically significant (p = 0.016) [12]. In the present study, among cases (hypomagnesemia patients), the mean range of duration of ICU stay was 6.12 days, and in the control group, it was 5.16 days. Kiran et al. reported that there was no statistically significant association between hypomagnesemia patients and an extended duration of ICU stay (p = 0.65), similar to the present study. In the present study, almost 48% of the patients in the case group needed invasive ventilator support, whereas only 28% of the patients in the control group required invasive ventilator support, which was statistically significant with a p-value of 0.03 [13]. Fiaccordori et al. reported that patients with lower muscle magnesium levels were on ventilatory support for a longer duration of time than those who had normal magnesium levels [14]. In the present study, we observed that the period of invasive ventilatory support was longer in the hypomagnesemia patients, with a mean of 10 days compared to three days in patients with normal magnesium levels, and this difference was statistically significant with a p-value of 0.001. Safavi et al. reported that in hypomagnesemia patients, the period of mechanical ventilation was prolonged with a mean of 7.2 days in cases compared to 4.7 days in the control group, which was statistically significant (p < 0.01) [15].

In the present study, we observed that decreased potassium levels were seen in 24% of cases and about 12% in controls, and this correlation was statistically insignificant with a p-value of 0.11. 12% of cases had hypocalcemia, compared to 8% in the controls group, and the correlation was statistically insignificant with a p-value of 0.11. Limaye et al. reported that approximately half of the patients (48%) with low potassium had low levels of serum magnesium, but the association was not statistically significant [16]. In the Ramesh Babu Panem et al. study, hypocalcemia was noticed in 54 out of 97 cases (55.97%), and the association between hypomagnesemia and hypocalcemia was statistically significant (p-value <0.05) [17]. Electrolyte abnormalities like hypokalemia, hypocalcemia, and hypophosphatemia are considered to be the predisposing factors for hypomagnesemia. In the present study, it was observed that mortality among hypomagnesemia patients was 28%, while in patients with normal levels of magnesium, it was 10%, which was statistically significant with a p-value of 0.02. Kiran et al. reported that the rate of mortality in hypomagnesemia cases was 51.1%, which was remarkably higher when compared to 36.2% in the normomagnesemic controls, and this difference was statistically significant [13]. Kongara et al. revealed that hypomagnesemia is associated with an increase in the mortality rate, and this correlation was statistically significant (p-value <0.05) [12].

Limitations

The small sample size is the major limitation. Other confounding factors, like chronic comorbidities, have not been eliminated. Large multi-centric trials need to be conducted to further strengthen the correlation.

## Conclusions

In hospitalized patients, hypomagnesemia is a common electrolyte abnormality, especially in critically ill patients. It has higher morbidity and mortality among critically ill patients. The need for invasive ventilation and duration of invasive ventilation were significantly higher among the patients with hypomagnesemia compared to the patients with normal magnesium levels (p-value <0.05). Mortality was higher in the cases than in the controls (p-value <0.05).
